# Accurate Sphingolipid Quantification Reducing Fragmentation Bias by Nonlinear Models

**DOI:** 10.1021/acs.analchem.3c02445

**Published:** 2023-10-02

**Authors:** Nina Troppmair, Dominik Kopczynski, Alice Assinger, Rainer Lehmann, Cristina Coman, Robert Ahrends

**Affiliations:** † Department of Analytical Chemistry, Faculty of Chemistry, University of Vienna, 1090 Vienna, Austria; ‡ Vienna Doctoral School in Chemistry, University of Vienna, 1090 Vienna, Austria; § Department of Vascular Biology and Thrombosis Research, Center of Physiology and Pharmacology, Medical University of Vienna, 1090 Vienna, Austria; ∥ Institute for Clinical Chemistry and Pathobiochemistry, Department for Diagnostic Laboratory Medicine, University Hospital Tuebingen, 72076 Tuebingen, Germany

## Abstract

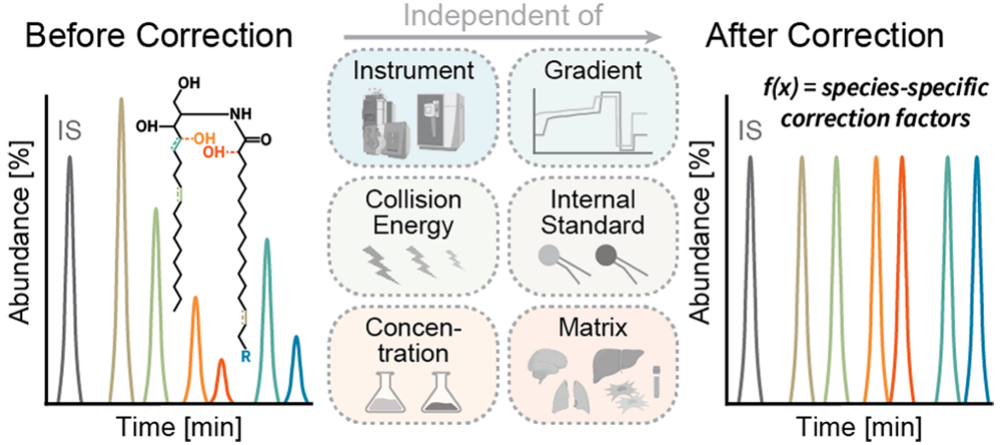

Quantitative sphingolipid analysis is crucial for understanding the roles of these bioactive molecules in various physiological and pathological contexts. Molecular sphingolipid species are typically quantified using sphingoid base-derived fragments relative to a class-specific internal standard. However, the commonly employed “one standard per class” strategy fails to account for fragmentation differences presented by the structural diversity of sphingolipids. To address this limitation, we developed a novel approach for quantitative sphingolipid analysis. This approach utilizes fragmentation models to correct for structural differences and thus overcomes the limitations associated with using a limited number of standards for quantification. Importantly, our method is independent of the internal standard, instrumental setup, and collision energy. Furthermore, we integrated this method into a user-friendly KNIME workflow. The validation results illustrate the effectiveness of our approach in accurately quantifying ceramide subclasses from various biological matrices. This breakthrough opens up new avenues for exploring sphingolipid metabolism and gaining insights into its implications.

## Introduction

As bioactive lipids and structural components, ceramides (Cer) are essentially involved in numerous cellular processes. They play important roles in differentiation, growth arrest, cellular senescence, apoptosis, and the formation of ceramide-rich platforms.^1−3^ Like other sphingolipids, ceramides are characterized by the presence of a sphingoid base (or long-chain base; LCB), as a structural backbone, on which an amide linked alkyl chain (fatty acid; FA) is attached.^2,4^ Different numbers and positions of double bonds and hydroxyl groups have been observed for the LCB, creating various sphingoid bases including dihydrosphingosine (e.g., 18:0;2), sphingosine (e.g., 18:1;2), phytosphingosine (e.g., 18:0;3), and 4,14-sphingadiene (or 4,8-sphingadiene; e.g., 18:2;2), with 18 carbon atoms being the prevalent chain length.^4,5^ The alkyl chain moieties vary in length, are mostly saturated or monounsaturated, and may have a hydroxyl group on the α- or ω-carbon atom.^1,4^ Overall, their structural diversity is underscored by a high degree of functional flexibility.^4^

To understand the function of ceramides in health or disease, thorough identification and quantification are required. This is commonly achieved by electrospray-based mass spectrometry (MS), which has become the method of choice in lipid and, therefore, ceramide analysis. Combining MS with liquid chromatography (LC) further improves the analysis reliability by reducing the number of overlapping lipid species.^6,7^ However, reversed-phase liquid chromatography (RPLC) often does not fully separate ceramide species. Thus, tandem mass spectrometry (MS/MS) with quantification on the MS^2^ level must be applied, and multiple fragments should be monitored.^8^ Quantitative studies require internal standards (IS) which compensate for variations in sample processing while serving as a reference for quantification.^9^ Ideally, isotope-labeled versions of the analytes of interest should be utilized as authentic ISs.^9,10^ However, using species-specific ISs is tedious and cost-intensive, and commercially available ISs do not encompass the extensive ceramide diversity. Alternatively, a single surrogate IS per lipid class can be used if specific requirements are met.^6,9^ The IS and analytes must be structurally similar to account for the different ionization efficiencies and ionize simultaneously.^6,11^ Still, even for the commonly used RPLC, these criteria are not fulfilled. The most frequently used lipid class ISs are structurally similar to the endogenously most abundant ceramides, but containing a non-naturally occurring FA.^6^ However, the analytical response of individual lipid species is not only determined by its lipid class but also by other structural elements like double bonds (DB), hydroxyl groups (OH), and chain length.^12−15^ Relying on a class-specific IS can, thus, give misleading quantitative results, especially for species structurally more differing from the IS. This results in functional misinterpretations as various ceramide subclasses that have distinct roles, e.g., in assembling or resolving lipid microdomains.^5^

Therefore, our goal was to develop a postacquisition correction model for this diverse family of lipids, ultimately allowing for their quantification utilizing one only class-specific IS. We investigated several ceramide subclasses to elucidate the influence of their chemical diversity on the instrument response, extending the commonly analyzed ceramides (see Supporting Information for nomenclature). The method has proven to be suitable for quantitative ceramide analysis in various biological samples with different instrumental setups and can be extended to further sphingolipids using a minimal number of standards.

## Experimental Section

### Materials

Chemicals for solvents were obtained from Sigma-Aldrich (Steinheim, Germany), Biosolve (Valkenswaard, The Netherlands), and Merck (Darmstadt, Germany). All sphingolipid standards were purchased from Avanti Polar Lipids (Alabaster, AL). More details are given in the Supporting Information.

### Sample Preparation

The herein developed correction method was applied to different biological samples, namely, mouse brain, mouse liver, mouse lung, OP9 cells, and human plasma. The mouse organs were homogenized by grinding in liquid nitrogen. The frozen organ was placed in a liquid nitrogen precooled ceramic mortar and manually ground until a fine powder was obtained. After grinding, the powder was collected into aliquots in precooled Eppendorf tubes. Four aliquots of each sample, OP9 cells, mouse tissue or human plasma, each containing approximately 300 μg of protein, were used for lipid extraction according to the protocol previously described by Coman et al.^16^ with minor adaptions (see Supporting Information for more details). Unless otherwise specified, Cer 18:1;2/12:0;0 was utilized as the IS for quantifying all ceramides.

### LC-MS/MS

Targeted ceramide analysis was performed as previously described by Peng et al.^8^ with minor optimizations using a Vanquish Flex UHPLC system (Thermo Fisher Scientific, Germering, Germany) coupled to a QTRAP 6500+ mass spectrometer (AB Sciex, Darmstadt, Germany). From each sample, 5 μL was injected onto an Ascentis Express C18 column (150 × 2.1 mm, 2.7 μm; Supelco, Bellefonte, PA) fitted with a guard cartridge (50 × 2.1 mm, 2.7 μm; Supelco, Bellefonte, PA) for chromatographic separation with the temperatures of the autosampler and the column oven set to 10 and 60 °C, respectively. Mobile phase A was ACN/H_2_O (60:40, v/v) and mobile phase B was IPA/ACN (90/10, v/v), both containing 10 mM ammonium formate, 0.1% formic acid, and 5 μM phosphoric acid. The 25 min long separation (details in Supporting Information) used a flow rate of 0.5 mL/min. Before each injection, the injector needle was automatically washed using 30% B with 0.1% phosphoric acid. The QTRAP system was equipped with an electrospray ion source (Turbo V ion source), and data were acquired in positive ion mode. Detailed information about source settings is provided in the Supporting Information. The collision energy (CE) was optimized for each ceramide standard. For the analysis in matrix applying scheduled multiple reaction monitoring (MRM), Q1 and Q3 were set to unit resolution, the detection window was set to 2 min, and the scan time was set to 0.5 s.

### Direct Infusion MS and MS/MS

For direct infusion experiments, the ceramide standards were dissolved in IPA/MeOH/chloroform (4:2:1, v/v/v) with 7.5 mM ammonium formate, and 12 μL of the sample was infused via TriVersa NanoMate ion source (Advion BioSciences, Ithaca, NY) into an Exploris 240 mass spectrometer (Thermo Fisher Scientific, Bremen, Germany). The detailed settings are described in the Supporting Information.

### Data Processing

Transition lists were calculated using LipidCreator (version 1.1.0.736).^17^ Data were acquired with Analyst (version 1.7.2; AB Sciex) for targeted ceramide analysis. Skyline (version 22.2.0.312)^18^ was used to visualize results and manually integrate signals. Processing of raw data from shotgun lipidomics experiments was performed using the R package rawrr^19^ in RStudio (version 2022.07.2 + 576; RStudio Team). Postacquisition data processing was automated using the software tool Konstanz Information Miner (KNIME; version 4.2.3).^20^ Subsequently, the quantities were corrected using the developed correction factors described herein. Figures were created with BioRender.com, OriginPro (Version 9.8.0.200; OriginLab Corporation), and ChemDraw (Version 20.0.0.41; PerkinElmer Informatics).

## Results and Discussion

### Challenges in Sphingolipid Analysis and Instrument Response of Ceramide Subgroups

**Figure 1:**
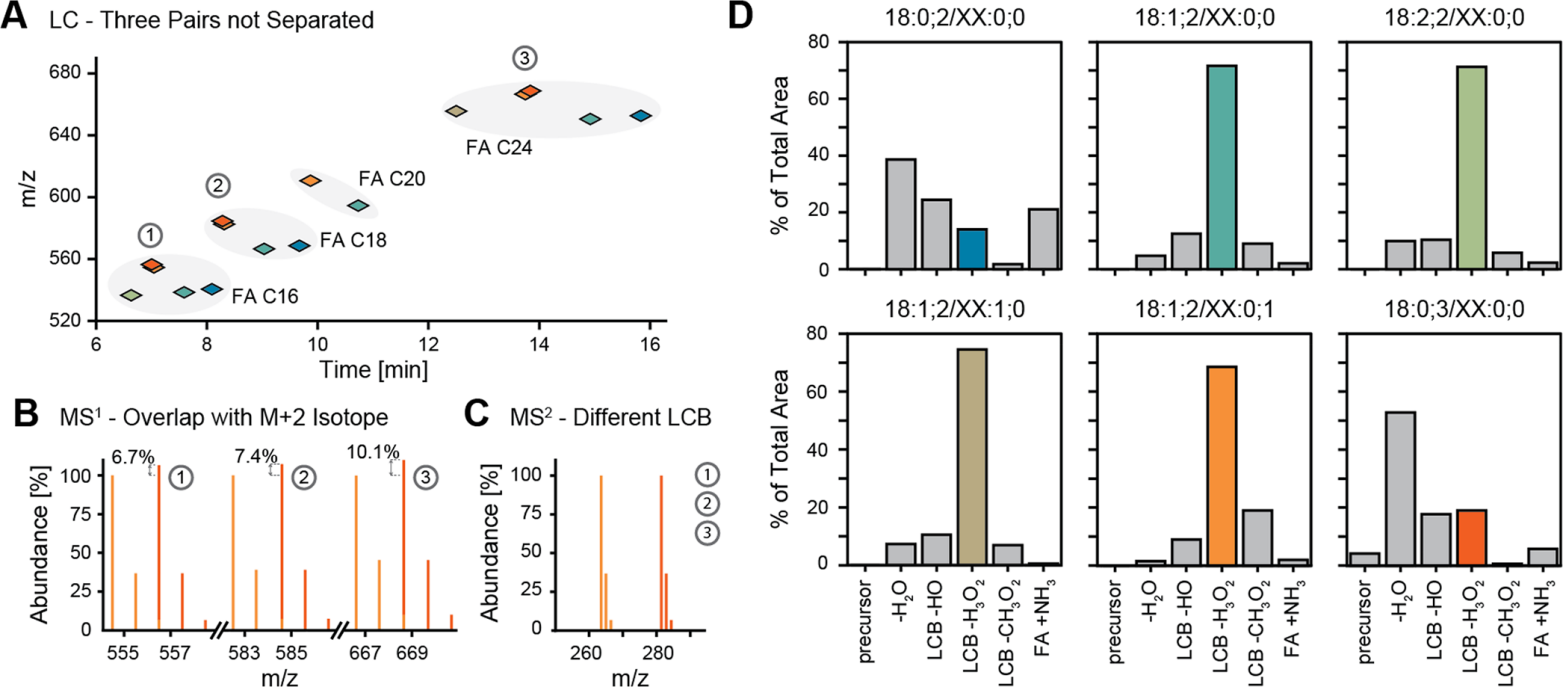
Challenges in sphingolipid analysis and main ceramide fragment types. (A) Based on their structure, ceramides elute at different time points of the gradient. As some species could not be chromatographically separated (①, ②, ③), the accurate quantification on MS1 level is hampered by the isotopic overlap of the monoisotopic signal and the second isotope of two species, overall differing by only one double bond (B). (C) Therefore, quantification on MS2 level using a fragment of the long-chain base (LCB) is necessary. (D) Ceramides show differences in their fragmentation pattern depending on certain structural features. Shown are the precursor and five fragments. Values are given in percent (%) of the summed areas of all analyzed transitions. The fragment used for quantification is highlighted in color.

Liquid chromatography electrospray ionization tandem mass spectrometry (LC-ESI-MS/MS) is the method of choice when aiming for comprehensive, in-depth profiling of sphingolipids due to its high level of specificity, sensitivity, and dynamic range. Besides others, the analysis of sphingolipids, and thus ceramides, is challenged by the presence of isobaric species, which might not be distinguishable by direct infusion MS.^4,8,21^ To mimic a real sample analysis, we have selected a set of standards (Figure 1A) which aims to summarize a few aspects of the ceramide diversity, and although limited by availability, our RPLC analysis pinpoints a common isobaric overlap of the second isotope with species differing only in the presence of one double bond (Figure 1B). In the case of the ceramide standards utilized in this work, this problem occurs for Cer 18:0;2/XX:0;0 and Cer 18:1;2/XX:0;0 or Cer 18:0;3/XX:0;0 and Cer 18:1;2/XX:0;1. With the method used, Cer 18:0;2/XX:0;0 and Cer 18:1;2/XX:0;0 can be chromatographically separated, which cannot be achieved for Cer 18:0;3/XX:0;0 and Cer 18:1;2/XX:0;1 (Figure 1A). Figure S1, as an extension of Figure 1A, further shows possible isomeric overlaps in addition to the isobaric ones. Since for all of these examples the LCB differs in more than only one double bond, a fragment of the LCB is well-suited to resolve this overlap and can be utilized for quantification, pointing to the benefits of quantifying on the MS^2^ level (Figure 1C). However, as previous studies on glycerophospholipids and steryl esters show, the molecule’s structure can influence the instrument response and readout.^13,15^ We observed that these differences in the fragmentation behavior were also substantial for ceramides by comparing the abundance of their fragments detected in MS/MS spectra (Figure 1D).

Besides the structural influence, the analyte response is influenced by multiple parameters, including ionization, solvent content, and sample composition.^9,22^ Keeping these constant within the lipid class of ceramides, the differences in structure-dependent fragmentation contribute considerably. To investigate the influence of the number and/or location of carbon atoms, double bonds, and hydroxyl groups of both the LCB and the FA, on the signal response, we analyzed 18 ceramide standards using LC-ESI-MS/MS. The respective structures are shown in Figure S2. In positive ion mode, ceramides are known to fragment by the loss of water and the FA, allowing the detection of different LCB fragments.^14,17^ We monitored six transitions of the protonated molecular ion to compare their intensities, covering the precursor, the water loss of the precursor, as well as three fragments belonging to the LCB pattern^8,23^ and a FA fragment. As depicted in Figure 1D, for Cer 18:1;2/XX:0;0, Cer 18:2;2/XX:0;0, Cer 18:1;2/XX:1;0, and Cer 18:1;2/XX:0;1, all having an unsaturated LCB, the most abundant fragment is LCB-H_3_O_2_; however, the saturated species Cer 18:0;2/XX:0;0 and Cer 18:0;3/XX:0;0 mostly fragment via the unspecific loss of water, resulting in an approximately 15-times higher signal than for the other studied ceramides. The LCB pattern commonly described for ceramides^8,14^ is only present for ceramides with an unsaturated LCB. The FA fragment is less frequent for most species, except for ceramides with a saturated LCB, for which it was observed at around 13 times higher intensity. Based on these observations, the intensities of the respective fragments, and in accordance with previously published methods on ceramide analysis,^10,24^ we chose to use the LCB-H_3_O_2_ fragment as a quantifier.

### Structure-Intensity-Relation of Different Ceramide Subgroups

**Figure 2:**
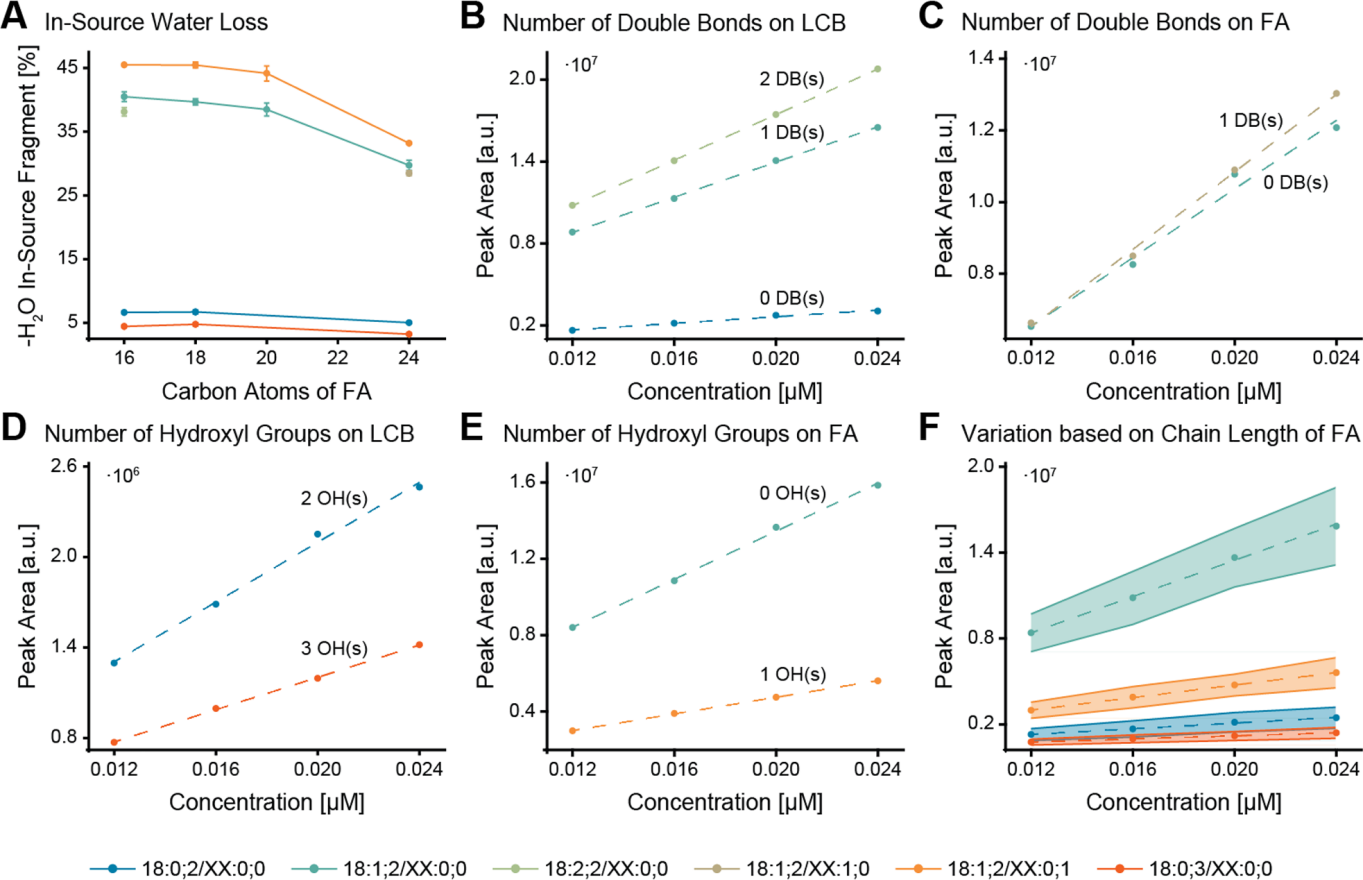
Structure-intensity-relation of long-chain base (LCB) fragment used for quantification. (A) In-source water loss of each analyzed ceramide subgroup; values are given in percent (%) of the summed areas of the precursor and water loss fragment. (B–F) Dilution series demonstrating the varying responses comparing specific structural features, namely, the number of double bonds on the LCB (B) and fatty acid (FA) (C), the number of hydroxyl groups on the LCB (D) and FA (E), as well as the FA chain length (F), respectively (with dashed regression lines and light areas showing the variation between across different FA chain lengths in (F)). The data is based on the analysis of four replicates.

Unsaturated ceramides are highly susceptible to losing a water molecule. Ceramides Cer 18:1;2/XX:0;0, Cer 18:2;2/XX:0;0, Cer 18:1;2/XX:1;0, and Cer 18:1;2/XX:0;1 exhibit eight times greater water loss than Cer 18:0;2/XX:0;0 and Cer 18:0;3/XX:0;0 (Figure 2A). Other in-source fragments account for roughly 1% of the summed areas of all fragments (Figure S3). Compared to different sphingolipid classes, ceramides show the highest degree of in-source fragmentation (Figure S4), which makes a response correction model particularly indispensable for this lipid class.

When comparing the LCB quantifier fragment across different subgroups with varying chain lengths, double bonds, and hydroxyl groups at different concentrations, significant differences in the signal response were observed depending on the degree of saturation and hydroxylation. Specifically, having no versus having a double bond on the LCB led to a more than 5-fold signal increase. In contrast, the second double bond had only a minor influence, resulting in a 20% increase in signal intensity (Figure 2B). An additional double bond on the FA did not significantly impact the quantifier intensity, as visible from the similar slopes (Figure 2C). However, the presence or absence of a hydroxyl group on the LCB or FA significantly influenced the signal intensity (Figure 2D, E). An additional hydroxyl group on the LCB or FA reduced the signal intensity by approximately a factor of 2, leading to low responses of Cer 18:0;3/XX:0;0 and Cer 18:1;2/XX:0;1. The influence of the chain length was less prominent (Figure 2F). The analytical response was slightly higher for smaller molecules as they have fewer degrees of freedom.^25^ This is in accordance with a previous study showing that Cer 18:1;2/16:0;0 has a higher response than Cer 18:1;2/18:0;0, which, in turn, has a higher response than 18:1;2/24:1;0.^14^ A model for the calculation of species-specific correction factors to correct for differences in the instrument response is thus inevitable for unbiased quantitative workflows.

The differences in response of ceramides belonging to the same subclass may be compensated for by selecting an average CE.^25^ However, this approach is not applicable when covering more diverse ceramides, as was monitored during this work. Comparing the CE of different species, shows that higher CEs are required for bigger molecules, which is supported by literature.^4^ Furthermore, molecules with fewer double bonds necessitate higher CEs. Studying the influence of hydroxyl groups, one must keep in mind that we were analyzing ceramides with an additional hydroxyl group on the LCB or on the FA. Looking at a fragment of the LCB, molecules with an additional hydroxyl group on the LCB fragment at a lower CE, while an additional hydroxyl group on the FA results in a higher CE necessary to cleave the bigger FA. Using the relation between the number of carbon atoms and the CE, the CE for other ceramides may be estimated. However, the optimization of the CE is not necessarily inevitable, as the error introduced by using the same CE for the entire lipid class is neglectable (in average below 10%) compared to the influence of the differences in fragmentation, which can lead to deviations of the actual concentration of up to 94 percentage points (Figure S5).

### Calculation of Correction Formula

The observations from the previously described experiments were used to determine a correction formula by using only one IS per lipid class. Within a subgroup, we observed a roughly linear relation between the length of the FA and the response. Thus, we started by calculating these linear equations for each of the subgroups. The error introduced by using a linear equation as an approximation is insignificant and can be disregarded (Figure S6). As the influence of the double bonds and hydroxyl groups is more complex and can only be described by a nonlinear model, we then determined and subsequently solved equations to calculate the respective parameters (Table S1). Finally, we designed the formula that can be used to correct for variations in the response of ceramides (Figure S7). Using the information on the double bonds, hydroxyl groups, and chain length, this approach allows us to accurately compute the response factor of ceramides of any subgroup studied in this work, ranging from 16 to 24 carbon atoms of the FA.

A KNIME^20^ workflow was developed to automate and accelerate data processing, including the application of correction factors. The workflow is described in greater detail in Figure S8. As the primary input, the workflow requires results from signal integration. Depending on the data set, the biological and technical replicates, the cell number or protein concentration for normalization, and the amount of the IS may be entered. Overall, the workflow has proven to be suitable for processing data from sphingolipid analyses of complex samples, giving the same quantification results as manually obtained from the same data set in 1/100 of the time.

### Model Evaluation

**Figure 3:**
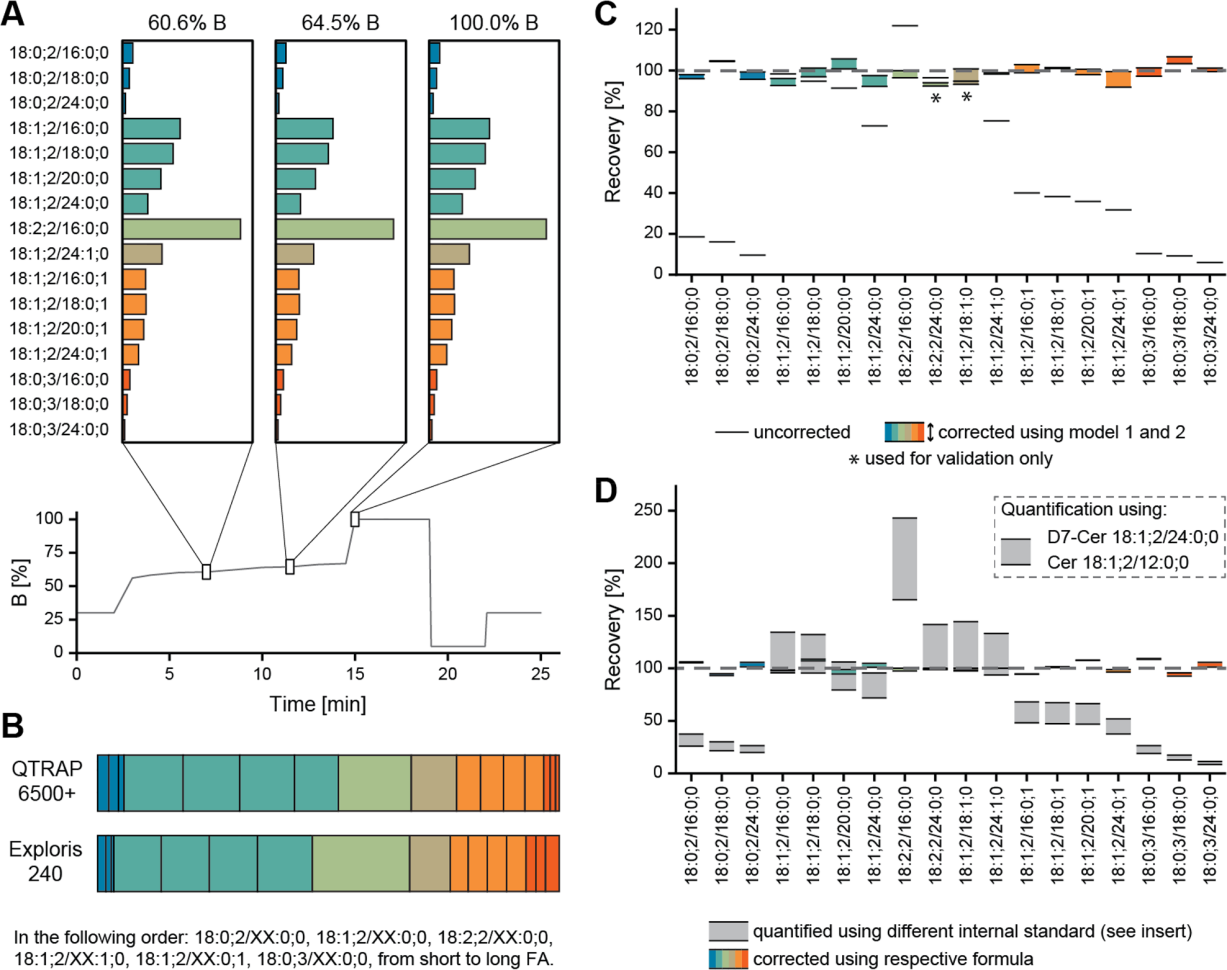
Evaluation of the developed formula for the calculation of correction factors. (A) Quantification dependency on the solvent composition. A mix of all tested ceramide species was analyzed without chromatographic separation, using the solvent composition at three time points along the gradient, respectively. (B) Comparison of the instrument response of the QTRAP 6500+ system coupled to a Vanquish Flex UHPLC system and the Orbitrap Exploris 240 equipped with a TriVersa NanoMate ion source. (C) Corrected and uncorrected recovery of tested ceramides comparing the determined correction formula to one developed by a second entirely computational approach. Cer 18:2;2/24:0;0 and Cer 18:1;2/18:1;0 were only used for testing the models but not when calculating the formula. (D) Recovery without and with correction when quantifying using Cer 18:1;2/12:0;0 and D7-Cer 18:1;2/24:0;0, respectively. Shown are the means of four replicate analyses.

After determining a formula for calculating factors to correct for the different responses of ceramide species, we aimed to evaluate whether our observations and factors were suitable for different setups. In the first step, we tested if changing the solvent composition during the LC gradient affected the different responses. To do so, we infused the ceramide standards using solvent compositions found at three different time points throughout the gradient, namely 60.6%, 64.5%, and 100.0% eluent B (Figure 3A). The intensities and ratios of the different species were constant and independent of the solvent, suggesting that the elution time during the gradient did not significantly influence the response and, therefore, the correction formula.

In addition to the QTRAP system, we analyzed the ceramide standards on a high-resolution orbitrap mass analyzer. Figure 3B shows the intensities of the quantifier ions of all tested ceramide subgroups normalized to 100%. The underrepresentation of species with a fully saturated LCB and FA was clearly present, as observed for the QTRAP, highlighting a similar structure-dependent fragmentation and an instrument independence of our observations. The major difference observed between the two instruments was the degree of in-source fragmentation, which was significantly lower on the Orbitrap Exploris 240 (Figure S9). As the relative reduction was similar among the tested ceramide species, the overall trends remained unchanged, allowing for the application of the correction model without further adaptions and therefore extending its applicability beyond the QTRAP.

Moreover, we validated all calculated correction factors using an alternative formula determined by a genetic computing method^26^ (Equation S1). This algorithm applies the strategy of selection, crossing, and mutation. The procedure is based on the same data set as the other approach, but we split the data into a training and a test set. We used the average relative residual (ARR) between the actual correction factor and the computed one as the optimization value. In the beginning, random equations are generated. In each iteration, half of the least-performing equations, with the highest ARR, are discarded. New equations are generated by crossing pairs of the remaining equations, and finally a subset of equations is randomly mutated by twiddling the coefficients and changing the arithmetic operation. The equation with the smallest errors has an ARR of 10.87 %. Comparing this model and the one previously described, the average deviation between the calculated correction factors is 3.25% (Figure 3C). In contrast, when using the average within each group of technical replicates, an optimal ARR of 8.38 % is achieved as a lower bound. This demonstrates the applicability of structure-based response factors for harmonizing the abundance of the LCB-H_3_O_2_ fragment between the ceramide species. Additionally, we analyzed Cer 18:2;2/24:0;0 and Cer 18:1;2/18:1;0, which were not included in the calculations for the formulas. With either of the two models, the corrected recovery is close to 100%, demonstrating the applicability to other ceramides within the included subgroups.

To demonstrate the adaptability of the final formula for the quantification using another IS, we quantified the tested ceramides using D7-Cer 18:1;2/24:0;0 as the IS (Figure 3D). Following the application of the correction, the error between the quantitative outcomes using Cer 18:1;2/12:0;0 and D7-Cer 18:1;2/24:0;0, respectively, averages less than 5%.

### Testing of the Model within Complex Matrices

It is well-known that compared to shotgun lipidomics, matrix effects can enhance or suppress signal intensities locally. This is particularly relevant if class-specific surrogate standards are used. To investigate whether matrix effects interfere with the application of the determined correction factors in the analysis of complex samples, we spiked different biological matrices with an equimolar mix of ceramide standards. The matrices were chosen based on their varying complexities, and we selected lipidomes with a tendency to complicate analysis due to their high glycerophospholipid (brain, lung), glycerolipid (liver, fat cells), and sterol content (plasma).^8,27^ Using standard addition, we determined the endogenous concentrations (for more details, see Supporting Information).

**Figure 4:**
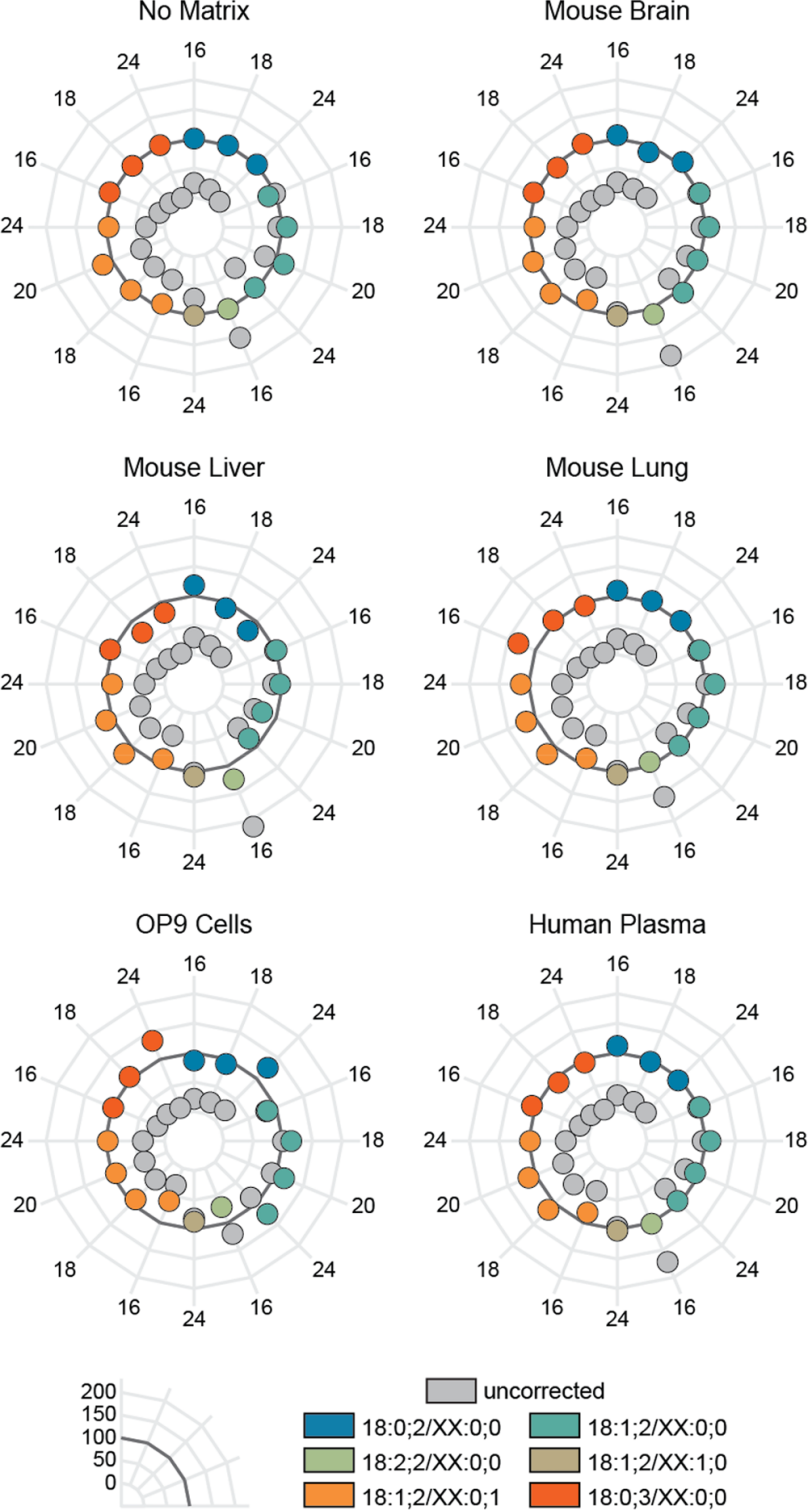
Recoveries of tested ceramides before and after correction in different matrices. The mix of ceramide standards was spiked into solvent or matrix (mouse brain, liver, lung, fat cells, and human plasma) to test if the formula is applicable to various biological samples. The dark line emphasizes 100% recovery, the gray circles represent the values without correction, and the colored ones show the corrected concentrations for each tested ceramide.

Our results show that the equimolar concentrations detected in biological samples, such as brain, liver, lung, fat cells, and plasma, correspond well with the trends observed for equimolar solutions in solvent without matrix (Figure 4). Among the studied ceramide species, Cer 18:1;2/16:0;0 gives the best recovery (calculated as the % of the known concentration in the samples) with the chosen IS, Cer 18:1;2/12:0;0, which was expected as it is structurally most similar. Comparing the corrected concentrations (colored circles) without and with matrix shows that due to the interference with other sample components, the mean deviation of the actual concentration in matrix is 10.72 percentage points, while without matrix it is only 4.44 percentage points. However, no trend in the deviation from the actual concentrations was observable, and the error is significantly lower than without correction (gray circles). The mean recovery without correction is 55.02%, with the lowest being below 5% of the actual concentration. In contrast, after applying the correction factors, the mean recovery in all tested matrices is 99.83%. These data demonstrate that the species-specific response factors determined herein are suitable for quantifying ceramides in various biological samples using only one IS for the entire lipid class.

**Figure 5:**
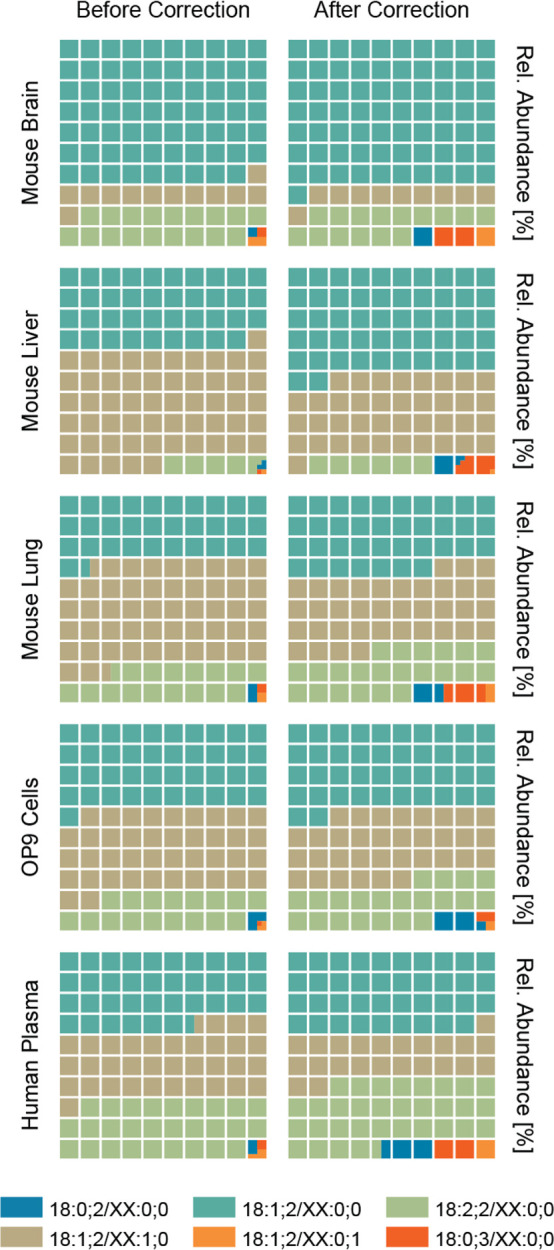
Ceramide quantities in tissues, cells and plasma without and with correction. For each of the six subgroups used throughout this work, the concentrations of ceramides having a fatty acid (FA) with 16, 18, and 24 carbon atoms were summed, and individual species were normalized to the sum. The distribution in different biological samples before and after the correction is visualized.

In mammals, the most abundant ceramides are those with the LCB 18:1;2 followed by 18:2;2.^28^ The FA varies more strongly in length and is usually saturated or monounsaturated.^3^ This is consistent with our observations, as shown in Figure 5, where we summed the concentrations of lipids with FA lengths of 16, 18, and 24 for each of the six groups of structurally related ceramides. As commonly known, Cer 18:1;2/XX:0;0, Cer 18:1;2/XX:1;0, and Cer 18:2;2/XX:0;0 are the major ceramides in mouse tissues, stem cells, and human plasma.^29,30^ While for the other subgroups the abundances of the different FA lengths are rather similar, Cer 18:1;2/24:1;0 is prevalent compared to species having a FA with 16 or 18 carbon atoms. Applying the correction presented herein to the endogenous ceramide concentrations demonstrates that some of the less prevalent ceramides are even more underestimated due to their poorer analytical response. The three least abundant subgroups of ceramides make up less than 1% in the uncorrected data but after the correction account for up to 5% of the ceramides monitored. Notably, these ceramides are involved in unique biological processes and cannot be replaced with ceramides with a different structure. For example, chain packing and thermal stability are significantly increased in lipid bilayer domains formed by saturated and hydroxylated ceramides. Also, gel phases formed by these ceramide species show higher transition temperatures, pointing to the role in membrane ordering and lipid interactions.^31^ Overall, this underscores the critical importance of unbiased quantification of all ceramide subclasses with various structural features to understand their signaling and the structural role in cellular biology.

### Method Validation

**Figure 6:**
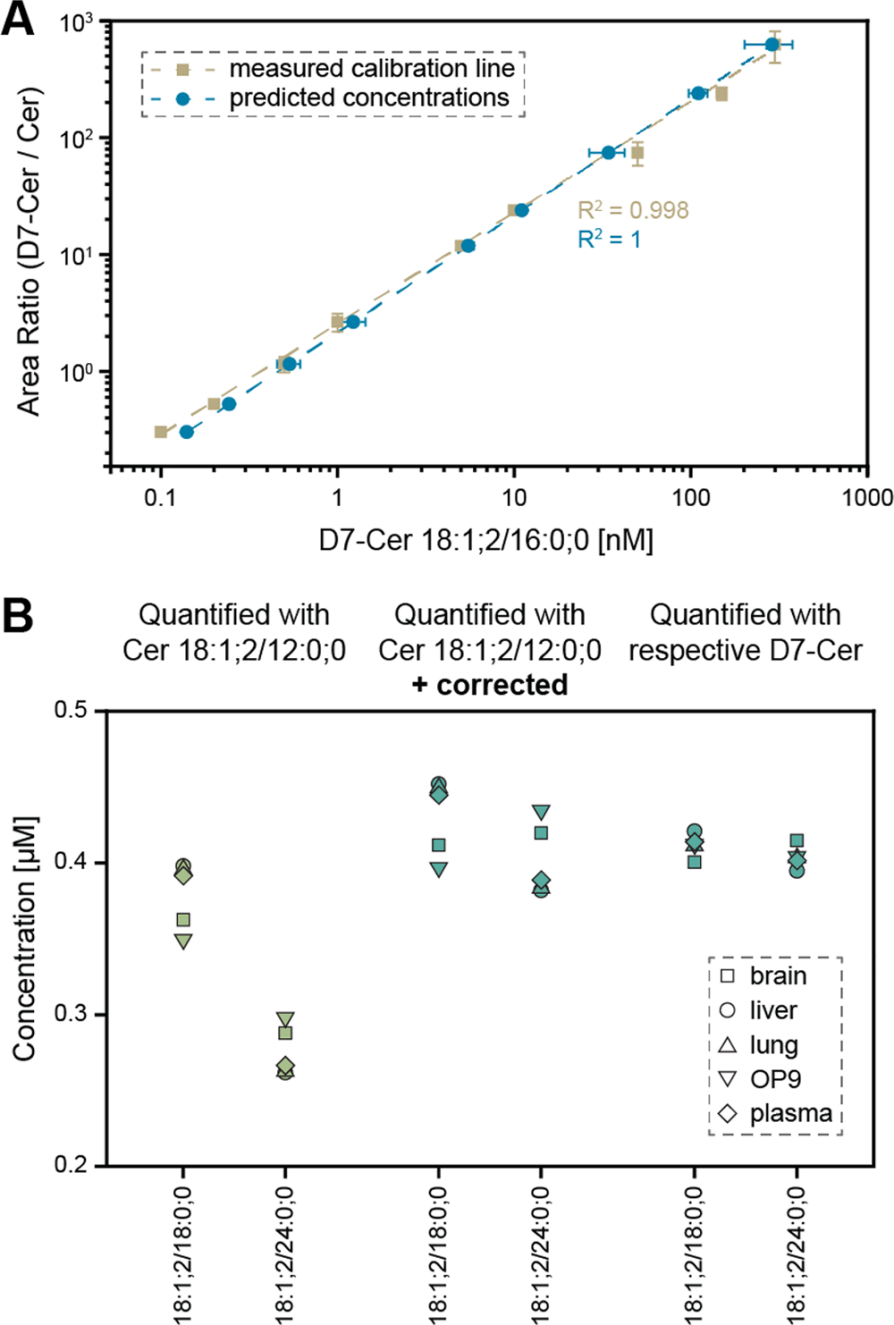
Validation of the quantitative results utilizing isotopically labeled standards. (A) Calibration curve and corrected concentrations. Mouse brain matrix was spiked with D7-Cer 18:1;2/16:0;0 at different concentrations. In addition, the concentration of D7-Cer 18:1;2/16:0;0 was calculated using the known concentration of Cer 18:1;2/12:0;0 and corrected by applying the respective correction factor. The mean and standard deviation of three replicates is shown. (B) Cer 18:1;2/18:0;0 and Cer 18:1;2/24:0;0 were quantified in mouse brain, liver, lung, fat cells, and human plasma. Both species were quantified using Cer 18:1;2/12:0;0, and subsequently, the quantitative data was corrected. In addition, the concentration was determined utilizing the isotope-labeled version of this specific species.

To demonstrate that our method can cover concentrations spanning more than 3 orders of magnitude, we spiked D7-Cer 18:1;2/16:0;0 into the mouse brain matrix at different concentrations, keeping the amount of Cer 18:1;2/12:0;0 constant. Subsequently, we assessed whether our model is feasible to cover the same concentration range as that previously determined. Thus, we calculated the concentration of D7-Cer 18:1;2/16:0;0 using Cer 18:1;2/12:0;0 for quantification and employed our model for correcting the differences in response. The calibration curve obtained through measurements and our model calculations are remarkably similar, underscoring the applicability of our model across a wide concentration range (Figure 6A). Additionally, we determined the ratios between the 18 ceramides measured at six different concentrations (Figure S10). The ratios remain constant across this concentration range, demonstrating that the correction factors can be applied without regard to the quantities.

Finally, by utilizing isotopically labeled standards, we aimed to validate the quantitative outcomes after applying the corrections in the different matrices. Initially, Cer 18:1;2/18:0;0 and Cer 18:1;2/24:0;0 were quantified using Cer 18:1;2/12:0;0. Subsequently, by applying the correction formula developed in this work, the quantitative data was corrected. To affirm the accuracy of this value, the quantity was determined by utilizing the isotope-labeled version of this specific species. Considering this latter approach as the most accurate,^7,9^Figure 6B underscores that implementing the correction for ceramide quantification yields comparable concentrations. It can further be seen that Cer 18:1;2/18:0;0 requires less correction compared to Cer 18:1;2/24:0;0, as it is structurally more similar to IS Cer 18:1;2/12:0;0.

The determined lower limit of detection (LLOD) and quantification (LLOQ) of the method are 0.03 and 0.06 nM, respectively. The intra- and interday precisions are given in Table S2.

## Conclusion

The functions of ceramides are diverse, as are their structure. Formed by distinct mechanisms in various compartments, up to 90 molecular species are known, differing in acyl chain length, saturation, and hydroxylation.^8,32,33^ Our understanding of the multifaceted roles of individual ceramide species is still in its infancy, and many questions remain to be answered. Unbiased ceramide species quantification allows for an improved delineation of the specific roles and functions of different pathways. By comprehensively assessing how structural features of ceramides influence their fragmentation behavior for the first time, a correction model was developed that significantly increases the quantification accuracy of different ceramide subclasses. We further demonstrated that dihydroceramides, 2′-hydroxy ceramides, and phytoceramides are usually underrepresented in our data sets, which may lead to misinterpretation of their biological impact.

The introduced approach proved to be fit for purpose, testing different matrices, CE, and instrument setups. The model provides the straightforward possibility for adaption to a different IS or more ceramide species, and to foster the usage of our correction model, we created a KNIME workflow that is freely accessible (upon request). In addition, the scheme presented herein can be extended to other sphingolipid classes if only a minimal set of surrogate standards is available.

In summary, this new approach for quantitative sphingolipid analytics offers a crucial tool for understanding the roles of sphingolipids in health and disease. The method is accurate and reliable and closes the gap between “one standard per class” and “one standard per species” quantification.

## Supporting Information Available

Lipid nomenclature, experimental procedures, additional data, supplemental equation, and references (PDF)
