# Supplementary material for: Accurate Sphingolipid Quantification Reducing Fragmentation Bias by Nonlinear Models
Source: Anal Chem. 2023 Oct 2;95(41):15227–35. doi: 10.1021/acs.analchem.3c02445 (PMC10585660; doi:10.1021/acs.analchem.3c02445)
Supplement: Supplementary file 1 — ac3c02445_si_001.pdf [file ac3c02445_si_001.pdf]

Supporting Information

Accurate Sphingolipid Quantification Reducing Fragmentation Bias by Nonlinear Models

Nina Troppmair[1,2], Dominik Kopczynski[1], Alice Assinger[3], Rainer Lehmann[4], Cristina Coman[1*], and Robert Ahrends[1*]

[1] Department of Analytical Chemistry, Faculty of Chemistry, University of Vienna, 1090 Vienna, Austria

[2] Vienna Doctoral School in Chemistry, University of Vienna, 1090 Vienna, Austria

[3] Department of Vascular Biology and Thrombosis Research, Center of Physiology and Pharmacology, Medical University of Vienna, 1090 Vienna, Austria

[4] Institute for Clinical Chemistry and Pathobiochemistry, Department for Diagnostic Laboratory Medicine, University Hospital Tuebingen, 72076 Tuebingen, Germany

*contributed equally

Table of contents

LIPID NOMENCLATURE 2
EXPERIMENTAL PROCEDURES 2
Text S1. Materials. 2
Text S2. Samples. 3
Text S3. Sample Preparation. 3
Text S4. LC-MS/MS. 3
Text S5. Direct Infusion MS and MS/MS. 3
Text S6. Internal standards and quantification. 4
Text S7. Method validation. 4
FIGURES 5
Figure S1. Isomeric overlaps of co-eluting ceramides. 5
Figure S2. Structures of the ceramide standards used for developing the correction model. 6
Figure S3. In-source fragments formed by the tested ceramides. 7
Figure S4. In-source fragmentation of other sphingolipid classes. 7
Figure S5. Collision energies of the tested ceramides. 8
Figure S6. Linear and quadratic fit to the measured correction factors. 8
Figure S7. Determining correction factors for ceramide quantification. 9
Figure S8. KNIME workflow for LC/MS-based analysis of ceramides in biological samples. 9
Figure S9. Difference in in-source fragmentation between QTRAP 6500+ and Exploris 240. 10
Figure S10. Concentration independence of response. 10
TABLES 11
Table S1. Parameters for correction formula. 11
Table S2. Intra- and Interday Precision. 11
EQUATIONS 12
Equation S1. Correction model determined for verification. 12
REFERENCES 12

LIPID NOMENCLATURE

For this work, we categorized ceramides extending the commonly known subclasses for different LCB backbones[1] by subgroups based on the saturation or hydroxylation of the FA. We used XX instead of the number of carbon atoms of the FA when pointing to all species of the subgroup, irrelevant of the FA length. The updated shorthand notation of sphingolipids[2] for example suggests annotating a ceramide with a LCB containing 18 carbon atoms, one double bond, and two hydroxyl groups with a N-linked FA with 16 carbon atoms and zero double bonds or hydroxyl groups as Cer 18:1;O2/16:0. However, for reasons of comparability and legibility we refer to this lipid as Cer 18:1;2/16:0;0, applying the nomenclature from Pauling *et al*.[3]

EXPERIMENTAL PROCEDURES

Text S1. Materials. Chemicals and lipid standards were obtained from the following sources: Ammonium formate, phosphoric acid, tert-butyl methyl ether (MTBE), and chloroform were obtained from Sigma-Aldrich (Steinheim, Germany). Acetonitrile (ACN), methanol (MeOH), water, and formic acid were purchased from Biosolve (Valkenswaard, The Netherlands), isopropanol (IPA), and 1-butanol (1-BuOH) from Merck (Darmstadt, Germany). All solvents were MS-grade. Sodium dodecyl sulfate (SDS) was obtained from Sigma-Aldrich (Steinheim, Germany), tris(hydroxymethyl)-aminomethane (Tris) from Roche Diagnostics (Mannheim, Germany), and sodium chloride (NaCl) from Merck (Darmstadt, Germany). Bicinchoninic acid (BCA) assay was purchased from Thermo Fisher Scientific (Rockford, IL). Ceramide/Sphingoid Internal Standard Mixture II (containing sphingosine 17:1;2, sphinganine 17:0;2, sphingosine-1-P 17:1;2, sphinganine-1-P 17:0;2, sphingomyelin 18:1;2/12:0;0, ceramide 18:1;2/12:0;0, glucosylceramide 18:1;2/12:0;0, lactosylceramide 18:1;2/12:0;0, and ceramide-1-P 18:1;2/12:0;0), mono-sulfo galactosyl(ß) ceramide 18:1;2/12:0;0, Deuterated Ceramide LIPIDOMIX® Mass Spec Standard (consisting of D7-ceramide 18:1;2/16:0;0, D7-ceramide 18:1;2/18:0;0, D7-ceramide 18:1;2/24:0;0, and D7-ceramide 18:1;2/24:1;0), ceramide 18:0;2/16:0;0, ceramide 18:0;2/18:0;0, ceramide 18:0;2/24:0;0, ceramide 18:1;2/16:0;0, ceramide 18:1;2/18:0;0, ceramide 18:1;2/20:0;0, ceramide 18:1;2/24:0;0, ceramide 18:2;2/16:0;0, ceramide 18:2;2/24:0;0, ceramide 18:1;2/18:1;0, ceramide 18:1;2/16:0;1, ceramide 18:1;2/18:0;1, ceramide 18:1;2/20:0;1, ceramide 18:1;2/24:0;1, ceramide 18:0;3/16:0;0, ceramide 18:0;3/18:0;0, and ceramide 18:0;3/24:0;0 were from Avanti Polar Lipids (Alabaster, AL).

Mouse mesenchymal stem cells (OP9 cells) were purchased from the Tokyo Metropolitan Institute of Medical Science. Minimum essential media (MEM-α), penicillin-streptomycin-glutamine (100x) solution, and phosphate buffered saline (PBS, pH = 7.4) were from Gibco (Bleiswijk, The Netherlands). Fetal bovine serum (FBS) was obtained from Sigma-Aldrich (Steinheim, Germany).

Text S2. Samples. Mouse organs were obtained from C57BL/6J mice, which were handled according to the ARRIVE guidelines with experiments approved by local authorities (BMBWF-V/3b/2023-0.184.589). After euthanization (ketamine (250 mg/kg) + xylazine (25 mg/kg)) of the mouse, the brain, liver, and lung were removed, snap-frozen in liquid nitrogen and stored at -80 °C. OP9 cells were cultured in a growth medium containing MEM-α, 2 mM L-glutamine, 20% FBS, 100 U/ml penicillin, and 100 U/ml streptomycin. The medium was changed every 2 days, cells were passaged every 4 days. Harvesting was performed by centrifugation, cells were washed with PBS and snap-frozen in liquid nitrogen prior to storage. For plasma samples, human blood collection by venipuncture into an EDTA (ethylenediaminetetraacetic acid) Sarstedt Monovettes (Nümbrecht, Germany) was approved by the local Ethics Committee of the medical faculty of Tübingen (113/2014BO1). The samples were mixed by gentle tilting and centrifuged at 3 800 g and 15 °C for 10 min. Subsequently, the top plasma layer was carefully pipetted out and immediately stored at −80 °C until further analysis.

Text S3. Sample Preparation. The lipid extraction was performed according to the protocol previously described by Coman *et al.*[4] with minor adaptions. In brief, 225 µl MeOH was added and the samples were homogenized by three consecutive freeze-thaw cycles, including ultrasonication (except plasma). Next, 750 µl MTBE and the internal standard (IS) mixture were added. After an incubation of 1 h at 950 rpm and 4 °C, 188 µl water was added to the samples to induce phase separation and the samples were centrifuged for 10 min at 4 °C and 10 000 g. Subsequently, the upper organic phase was carefully collected and dried under nitrogen flow. The dried lipids were reconstituted in 1-BuOH/IPA/H_2O (8:23:69, v/v/v) containing 5 mM phosphoric acid.[5] For protein precipitation, 527 µl MeOH was added to the lower aqueous phase and the samples were stored at -20 °C for 2 h prior to centrifugation for 30 min at 13 500 g. The supernatant was removed, and after drying the pellet, proteins were dissolved in 1% SDS, 150 mM NaCl, and 50 mM Tris (pH 7.8) and quantified based on the BCA assay.

Text S4. LC-MS/MS. The following gradient was applied: initial 30.0% B, held at 30.0% B from 0.0 to 2.0 min, 56.1% B at 3.0 min, 58.3% B at 4.0 min, 60.2% B at 5.5 min, 60.6% B at 7.0 min, 62.3% B 8.5 min, 64.0% B 10.0 min, 64.5% B 11.5 min, 66.2% B at 13.0 min, 66.9% B at 14.5 min, 100.0% B from 15.0 to 19.0 min, 5.0% B from 19.1 to 22.0 min, and 30 % B from 22.1 to 25.0 min.

The ESI source settings were the following: curtain gas 30 psi, collision gas medium, ion spray voltage +5500 V, temperature 250 °C, ion source gas 1 40 psi, ion source gas 2 65 psi, declustering potential +75 V, entrance potential +10 V, and exit potential +17 V.

Text S5. Direct Infusion MS and MS/MS. The ionization voltage was set to +1.25 kV in positive and –1.25 kV in negative mode. The back pressure was 0.95 psi in both polarities, the temperature of the ion transfer capillary was 250 °C, and the s-lens level was 60%. The 'Mild Trapping' option was selected

to reduce unintentional dissociation of the precursor ion. Full MS spectra covering the mass range from 400 – 750 m/z in positive mode and 400 – 800 m/z in negative mode were acquired with a resolution of 240 000, an AGC target of 10^6, and a maximum IT of 105 ms. MS/MS spectra were acquired with a resolution of 60 000, an AGC target of 10^5, and a maximum IT of 105 ms. The isolation window was 1 m/z and the nCE was 27% and 30% for positive and negative mode, respectively.

Text S6. Internal standards and quantification. Exclusively Cer 18:1;2/12:0;0 was employed as IS, aligning with the objective of this work to develop a correction model for the quantification of ceramides using only one IS for the entire class. The added amount of the IS was selected to be in an appropriate ratio to the analytes investigated in the biological matrices, considering their dynamic range. We have selected Cer 18:1;2/12:0;0 as a convenient non-naturally occurring species and proved its applicability in comparison to D7-Cer 18:1;2/24:0;0 which elutes later in our separation (Figure 3D). This data also demonstrates that by using our correction model one standard per class is sufficient for accurate quantitation.

For testing the correction formula in matrix, the samples were extracted without the addition of any IS. The IS Cer 18:1;2/12:0;0 was spiked into the samples together with the other standards after the extraction to avoid causing differences in the determined quantities due to differing extraction efficiencies. The concentration was determined using the principle of standard addition. The mean of four measurements of the matrix and spiked samples (0.2 and 0.4 µM) was used to calculate the endogenous concentration in the samples.

For applying the correction model to the endogenous levels, each of the samples was extracted again, adding the IS in the beginning of samples preparation, as commonly recommended.[6, 7] Each matrix was extracted in four replicates and measured separately and then pooled for the final data.

As the isotope-labeled analog of the analyte of interest is considered the ideal IS[6, 8], the isotope-labeled ISs D7-Cer 18:1;2/16:0;0, D7-Cer 18:1;2/18:0;0, and D7-Cer 18:1;2/24:0;0 were employed to validate the quantitative results.

The most abundant adduct observed for ceramides in positive mode is the protonated molecular ion, followed by the sodium adduct, accounting for an average of 14% of the protonated one. In negative mode, with the herein used mobile phases, the formate adduct is more abundant than the deprotonated molecular ion. As overall, the protonated molecular ion gives the highest signal, only this adduct will be considered throughout this work.

Text S7. Method validation. In addition to the extensively discussed accuracy of ceramide quantification, the applied method was validated for its linearity, sensitivity, precision, and dilution integrity based on the ICH guideline.[9]

Linearity. To establish a calibration curve, we spiked varying concentrations of D7-Cer 18:1;2/16:0;0 into the mouse brain matrix, keeping the concentration of Cer 18:1;2/12:0;0 constant (n=3). In addition to nine calibration standards (0.1, 0.2, 0.5, 1, 5, 10, 50, 150, and 300 nM). Subsequently, we assessed whether our model is feasible to cover the same concentration range as previously determined. To do so, we calculated the concentration of D7-Cer 18:1;2/16:0;0 using Cer 18:1;2/12:0;0 for quantification and subsequently employed our model for correcting the differences in response (Figure 6A).

Sensitivity. The LLOD and LLOQ were determined by calculating the signal-to-noise ratios (S/N) at the two lowest concentrations of the calibration curve (0.1 and 0.2 nM). The S/N values were determined using Analyst (version 1.7.2; AB Sciex). As commonly defined, a ratio of 3:1 was considered the LLOD, while a ratio of 5:1 was used as the LLOQ.

Precision. To evaluate the method's reproducibility, we utilized OP9 cells as the matrix and spiked them with Cer 18:1;2/12:0;0. The intraday precision was evaluated by analyzing the samples four times on the same day, while the interday precision was determined by conducting the analysis on four different days. The precision of both normalized area and retention time, was determined using the percent coefficient of variation (CV) (Table S2).

Dilution integrity. To show that dilution of the sample does not impact the accuracy and precision of the measurements, we analysed a mixture of the eighteen ceramides at six different concentrations (0.5, 5, 10, 20, 25, 50 nM) (Figure S10).

FIGURES

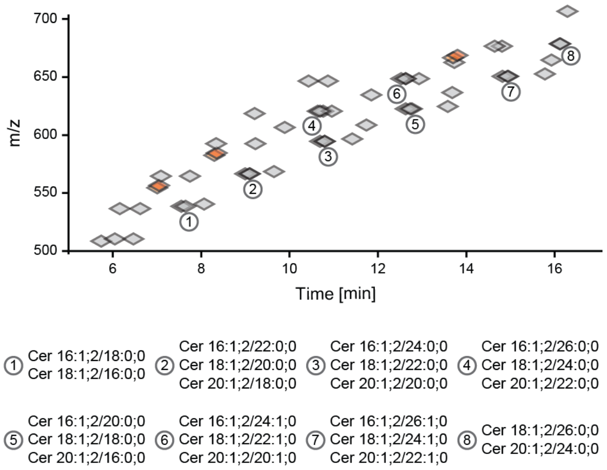

Figure S1. Isomeric overlaps of co-eluting ceramides. As an extension to Figure 1A, this shows additional ceramides typically identified in the herein used matrices. In addition to the isobaric overlaps arising from the second isotope of species differing only in the presence or absence of a double bond, there are eight isomeric overlaps. These are species only varying in their chain lengths and, thus, cannot be differentiated using only MS1 information.

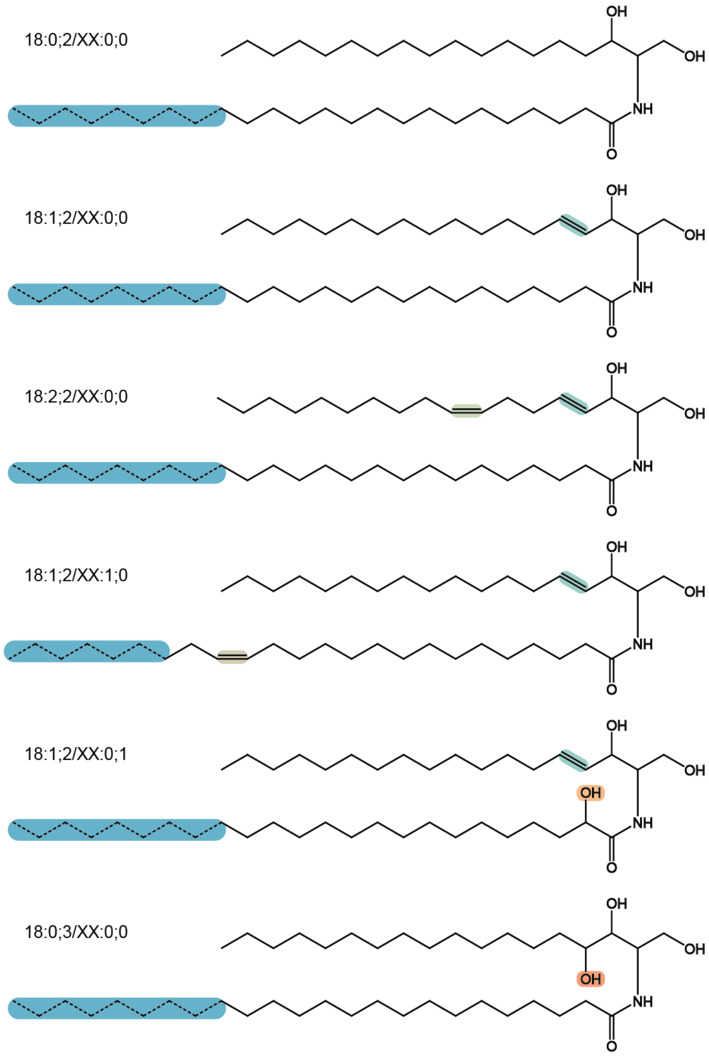

Figure S2. Structures of the ceramide standards used for developing the correction model. The molecules differ in chain lengths, double bonds, and hydroxyl groups. For the subclasses Cer 18:2;2/XX:0;0 and Cer 18:1;2/XX:1;0 the position of the additional double bond corresponds to the one of the species used for calculating the formula.

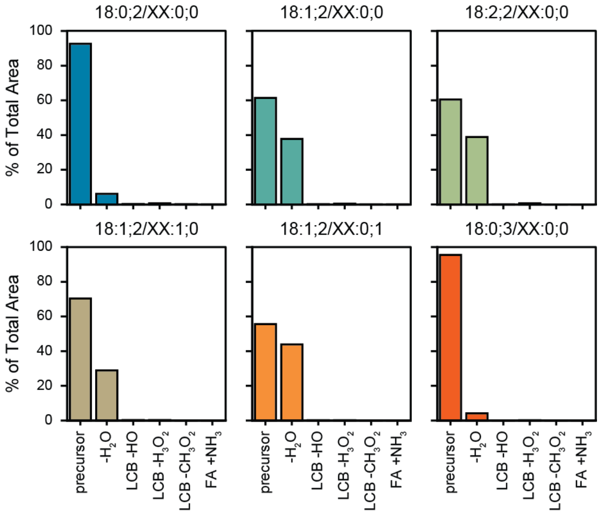

Figure S3. In-source fragments formed by the tested ceramides. Besides the water-loss no fragments are formed in the source at a significant level. Shown are the precursor and five fragments. Values are given in percent (%) of the summed areas of all analyzed transitions. For the subclasses Cer 18:0;2/XX:0;0, Cer 18:1;2/XX:0;0, Cer 18:1;2/XX:0;1, and Cer 18:0;3/XX:0;0 the mean of the different chain lengths was calculated.

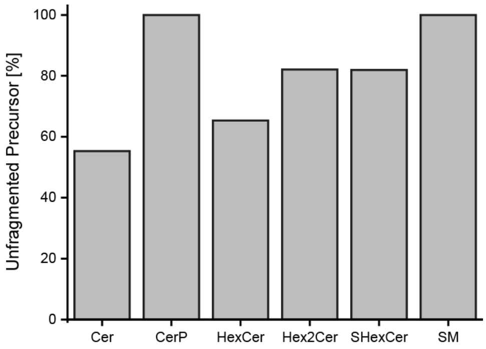

Figure S4. In-source fragmentation of other sphingolipid classes. Shown is the relative quantity of the molecule not fragmented in the source. Ceramides are more prone to in-source fragmentation than sphingolipids belonging to other classes, namely ceramide-1-P (CerP), glucosylceramide (HexCer), lactosylceramide (Hex2Cer), mono-sulfo galactosyl(ß) ceramide (SHexCer), and sphingomyelin (SM).

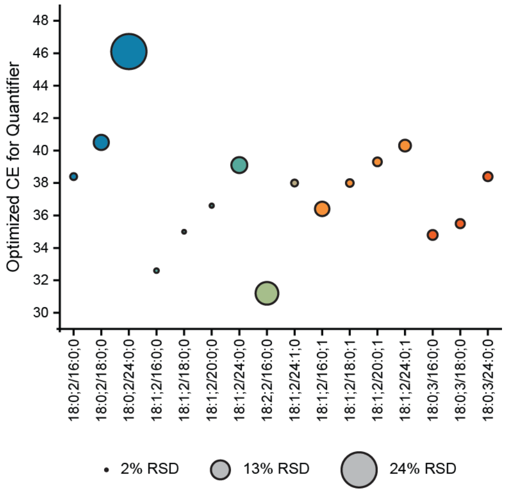

Figure S5. Collision energies of the tested ceramides. The optimized collision energy (CE) for the quantifier fragment LCB-H_3O_2 is shown for every species. The size of the circles displays the relative standard deviation (RSD) of the integrated signals using a CE of 38, 39, 40, 41, 42, and the optimized one on the QTRAP 6500+. So, even testing this wide range of CE, the average error resulting from the different fragmentation patterns is higher than the one caused by a non-optimized CE. This suggests that an CE optimization is not necessarily essential, but a common CE for the entire lipid class may be chosen.

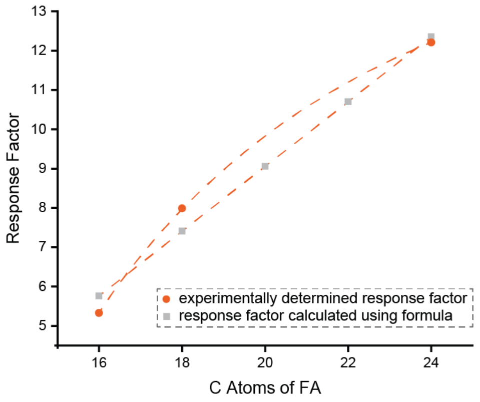

Figure S6. Linear and quadratic fit to the measured correction factors. The subgroup Cer 18:0;3/XX:0;0 is shown as an example. A quadratic equation would better describe the observed response than a linear one, however, the linear equation serves as an approximation, simplifying the formula for ease of use. The disparity between the linear and quadratic equations is minor and can be disregarded, as the chain length has a minimal impact compared to the presence of double bonds and hydroxyl groups. The mean relative error between the response factors calculated using linear and quadratic fitting is only 2.9% for all ceramides covered by this formula.

Step 1: For each subgroup of ceramides differing only in the length of the FA (same number and position of double bonds and hydroxyl groups on both), we computed linear equations with parameters m (slope) and b (intercept) for calculating the correction factors, using the number of carbon atoms of the FA as variable.

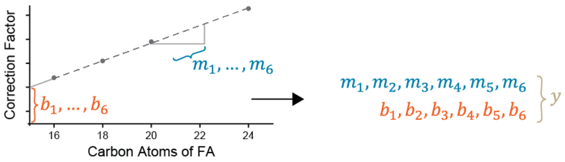

Step 2: We determined equations describing the quadratic relation between the previously calculated parameters and the double bonds and hydroxyl groups on the LCB and FA, respectively.

$$\begin{bmatrix} DB_{LCB,1} & OH_{LCB,1} & DB_{FA,1} & OH_{FA,1} & (DB_{LCB,1}+DB_{FA,1})^2 & (OH_{LCB,1}+OH_{FA,1})^2 \\ \vdots & \vdots & \vdots & \vdots & \vdots & \vdots \\ DB_{LCB,6} & OH_{LCB,6} & DB_{FA,6} & OH_{FA,6} & (DB_{LCB,6}+DB_{FA,6})^2 & (OH_{LCB,6}+OH_{FA,6})^2 \end{bmatrix} \begin{bmatrix} p_1 \\ \vdots \\ p_6 \end{bmatrix} = \begin{bmatrix} m_1 \\ \vdots \\ m_6 \end{bmatrix}$$

$$\begin{bmatrix} DB_{LCB,1} & OH_{LCB,1} & DB_{FA,1} & OH_{FA,1} & (DB_{LCB,1}+DB_{FA,1})^2 & (OH_{LCB,1}+OH_{FA,1})^2 \\ \vdots & \vdots & \vdots & \vdots & \vdots & \vdots \\ DB_{LCB,6} & OH_{LCB,6} & DB_{FA,6} & OH_{FA,6} & (DB_{LCB,6}+DB_{FA,6})^2 & (OH_{LCB,6}+OH_{FA,6})^2 \end{bmatrix} \begin{bmatrix} q_1 \\ \vdots \\ q_6 \end{bmatrix} = \begin{bmatrix} b_1 \\ \vdots \\ b_6 \end{bmatrix}$$

Step 3: We solved the system of equations (see y and x summarizing m and b as well as p and q, respectively, to demonstrate the operations) using R, obtaining parameters p and q to calculate the slope and intercept for any included ceramide species.

$$\mathbf{A} \cdot \vec{x} = \vec{y}$$

$$\vec{x} = \mathbf{A}^{-1} \cdot \vec{y}$$

$$x \begin{cases} p_1, p_2, p_3, p_4, p_5, p_6 \\ q_1, q_2, q_3, q_4, q_5, q_6 \end{cases}$$

Step 4: Based on the species' structure, the final equation enables the correction of ceramide quantities from any subgroup covered in this work.

$$correction\ factor = C_{FA} \cdot (p_1 \cdot DB_{LCB} + p_2 \cdot OH_{LCB} + p_3 \cdot DB_{FA} + p_4 \cdot OH_{FA} + p_5 \cdot (DB_{LCB} + DB_{FA})^2 + p_6 \cdot (OH_{LCB} + OH_{FA})^2) + q_1 \cdot DB_{LCB} + q_2 \cdot OH_{LCB} + q_3 \cdot DB_{FA} + q_4 \cdot OH_{FA} + q_5 \cdot (DB_{LCB} + DB_{FA})^2 + q_6 \cdot (OH_{LCB} + OH_{FA})^2$$

Figure S7. Determining correction factors for ceramide quantification. The formula was determined in four steps, starting with finding linear equations to describe the influence of the fatty acid (FA) length, determining and subsequently solving equations to calculate these parameters and in the end formulating the final formula, which may be used to compute the correction factor of a ceramide species based on the FA length, double bonds (DB) and hydroxyl groups (OH) of the long chain base (LCB) and FA, respectively.

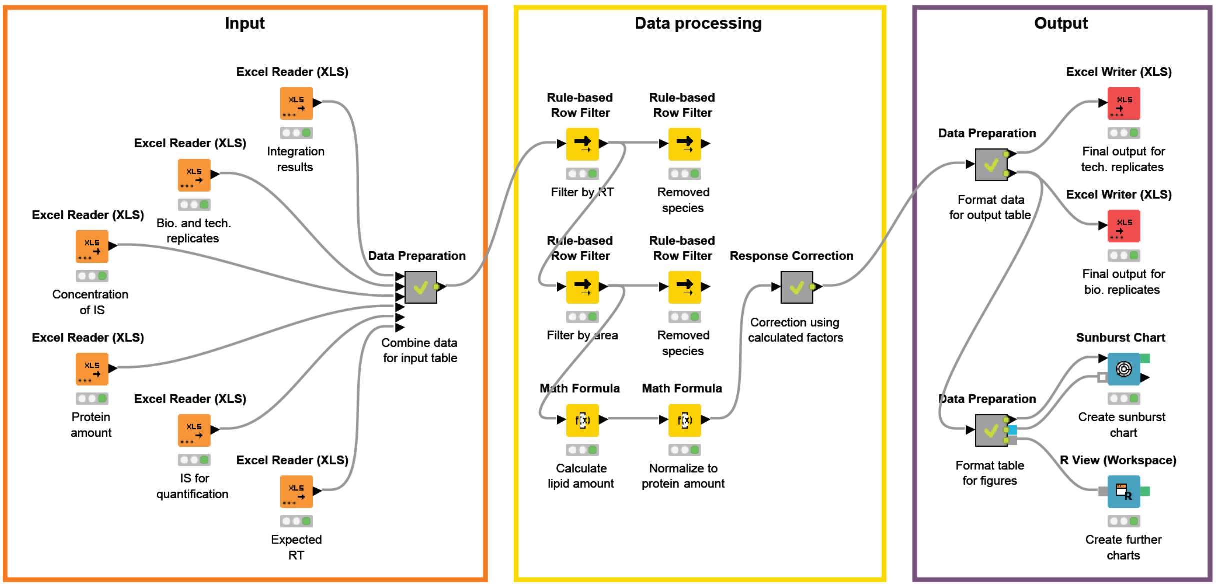

Figure S8. KNIME workflow for LC/MS-based analysis of ceramides in biological samples. The developed KNIME workflow supports the comprehensive analysis of ceramides in samples, calculating the lipid concentration in the samples using correction factors. As input the workflow requires the integrated data, the respective biological and technical replicates, internal standards (IS) assigned to the endogenous lipids, the amount of the IS, the protein concentration for normalization, and the expected retention times (RT) if non-fitting species should be excluded. The orange boxes depict these necessary input tables. The yellow boxes show the data processing, yielding output tables, displayed as red boxes, as well as several graphs, represented by blue boxes. To keep the workflow more compact, some nodes were grouped into so-called meta-nodes, symbolized by grey boxes.

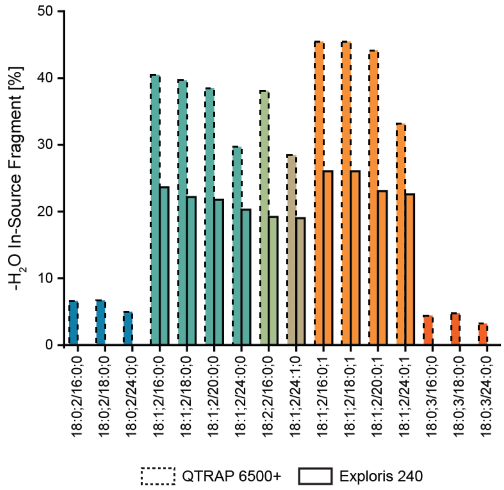

Figure S9. Difference in in-source fragmentation between QTRAP 6500+ and Exploris 240. For all ceramides the in-source fragmentation was significantly lower on the Exploris. However, the trends between the different ceramide species are not altered, so the correction model may be applied without adaptions.

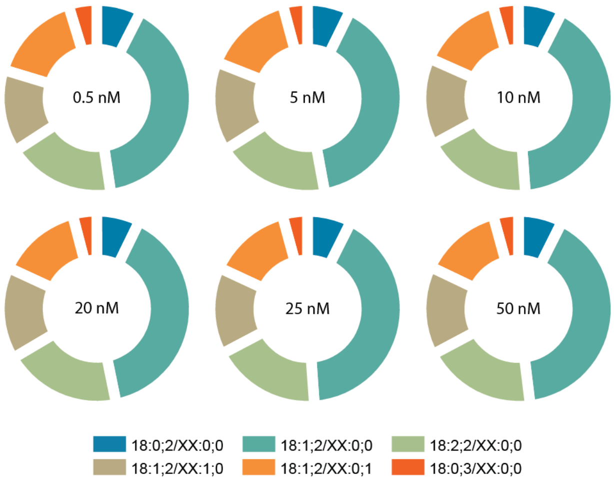

Figure S10. Concentration independence of response. Ratios between the six ceramide subgroups measured at different concentrations. As the ratios remain constant across, the correction factors can be applied without regard to the concentration.

TABLES

Table S1. Parameters for correction formula. The following parameters of the herein described correction formula (correction factor = $C_{FA} \cdot (p_1 \cdot DB_{LCB} + p_2 \cdot OH_{LCB} + p_3 \cdot DB_{FA} + p_4 \cdot OH_{FA} + p_5 \cdot (DB_{LCB} + DB_{FA})2 + p6 \cdot (OH_{LCB} + OH_{FA})^2) + q_1 \cdot DB_{LCB} + q_2 \cdot OH_{LCB} + q_3 \cdot DB_{FA} + q_4 \cdot OH_{FA} + q_5 \cdot (DB_{LCB} + DB_{FA})2 + q_6 \cdot (OH_{LCB} + OH_{FA})^2$) were calculated for the IS and instrumental setup applied. If any of these are adapted, the parameters may be recalculated straightforwardly.

Parameter	Value
p_1	-0.15310
p_2	-0.31423
p_3	-0.19242
p_4	-0.95186
p_5	0.050341
p_6	0.19624
q_1	-1.8480
q_2	7.2456
q_3	-0.84211
q_4	16.656
q_5	0.48123
q_6	-3.2389

Table S2. Intra- and Interday Precision. OP9 cell matrix was spiked with the 18 herein used ceramide standards and the sample was analyzed four times on the same and different day(s). The precision was determined for the normalized area and the retention time.

	Normalized area [a.u.]				Retention time [min]			
	Intraday		Interday		Intraday		Interday	
Species	Mean	CV (%)	Mean	CV (%)	Mean	CV (%)	Mean	CV (%)
Cer 18:0;2/16:0;0	0.10	5.1	0.12	4.5	8.4	0.0	8.4	0.1
Cer 18:0;2/18:0;0	0.061	5.8	0.064	7.3	10	0.0	10	0.1
Cer 18:0;2/24:0;0	0.064	6.6	0.063	7.8	16	0.0	16	0.0
Cer 18:1;2/16:0;0	6.1	2.4	6.1	2.6	7.9	0.0	7.8	0.3
Cer 18:1;2/18:0;0	0.48	2.0	0.48	4.7	9.4	0.1	9.3	0.2
Cer 18:1;2/20:0;0	0.34	4.9	0.33	1.6	11	0.0	11	0.2
Cer 18:1;2/24:0;0	3.0	4.4	3.0	7.3	16	0.1	15	0.2
Cer 18:2;2/16:0;0	0.97	1.1	0.94	3.8	6.8	0.2	6.8	0.0
Cer 18:2;2/24:0;0	0.20	3.3	0.19	3.0	14	0.0	14	0.1
Cer 18:1;2/18:1;0	0.33	1.6	0.34	4.5	8.0	0.2	8.0	0.0
Cer 18:1;2/24:1;0	0.19	1.2	0.19	3.4	13	0.1	13	0.1
Cer 18:1;2/16:0;1	0.14	4.6	0.15	7.8	7.2	0.1	7.2	0.1
Cer 18:1;2/18:0;1	0.12	5.2	0.13	2.7	8.6	0.0	8.6	0.1
Cer 18:1;2/20:0;1	0.098	5.4	0.099	3.4	10	0.1	10	0.2
Cer 18:1;2/24:0;1	0.084	2.2	0.083	10	14	0.1	14	0.1
Cer 18:0;3/16:0;0	0.071	2.5	0.072	10	7.2	0.0	7.2	0.2
Cer 18:0;3/18:0;0	0.051	4.9	0.052	5.8	8.6	0.0	8.5	0.2
Cer 18:0;3/24:0;0	0.029	11	0.029	11	14	0.1	14	0.2

EQUATIONS

Equation S1. Correction model determined for verification. This formula was determined using a genetic computing method. The average deviation between this and the final formula is less than 5%.

$$correction\,factor = \frac{\frac{DB_{LCB}\ \cdot 29.08 - (OH_{LCB} + 12.30 - (C_{FA} + DB_{FA}) \cdot OH_{LCB})}{4.923}}{0.6792\ +\ DB_{LCB} \cdot 9.403} + \frac{OH_{FA}}{0.6137}$$

REFERENCES

(1) Fahy, E.; Subramaniam, S.; Brown, H. A.; Glass, C. K.; Merrill, A. H., Jr.; Murphy, R. C.; Raetz, C. R. H.; Russell, D. W.; Seyama, Y.; Shaw, W.; et al. A comprehensive classification system for lipids. *Journal of Lipid Research* 2005, *46* (5), 839-861. DOI: 10.1194/jlr.E400004-JLR200.

(2) Liebisch, G.; Fahy, E.; Aoki, J.; Dennis, E. A.; Durand, T.; Ejsing, C. S.; Fedorova, M.; Feussner, I.; Griffiths, W. J.; Köfeler, H.; et al. Update on LIPID MAPS classification, nomenclature, and shorthand notation for MS-derived lipid structures. *Journal of Lipid Research* 2020, *61* (12), 1539-1555. DOI: 10.1194/jlr.S120001025.

(3) Pauling, J. K.; Hermansson, M.; Hartler, J.; Christiansen, K.; Gallego, S. F.; Peng, B.; Ahrends, R.; Ejsing, C. S. Proposal for a common nomenclature for fragment ions in mass spectra of lipids. *PLOS ONE* 2017, *12* (11). DOI: 10.1371/journal.pone.0188394.

(4) Coman, C.; Solari, F. A.; Hentschel, A.; Sickmann, A.; Zahedi, R. P.; Ahrends, R. Simultaneous Metabolite, Protein, Lipid Extraction (SIMPLEX): A Combinatorial Multimolecular Omics Approach for Systems Biology. *Molecular & cellular proteomics* 2016, *15* (4), 1435-1466. DOI: 10.1074/mcp.M115.053702.

(5) Danne-Rasche, N.; Coman, C.; Ahrends, R. Nano-LC/NSI MS Refines Lipidomics by Enhancing Lipid Coverage, Measurement Sensitivity, and Linear Dynamic Range. *Analytical Chemistry* 2018, *90* (13), 8093–8101. DOI: 10.1021/acs.analchem.8b01275.

(6) Wang, M.; Wang, C.; Han, X. Selection of internal standards for accurate quantification of complex lipid species in biological extracts by electrospray ionization mass spectrometry-What, how and why? *Mass spectrometry reviews* 2017, *36* (6), 693-714. DOI: 10.1002/mas.21492.

(7) Burla, B.; Arita, M.; Arita, M.; Bendt, A. K.; Cazenave-Gassiot, A.; Dennis, E. A.; Ekroos, K.; Han, X.; Ikeda, K.; Liebisch, G.; et al. MS-based lipidomics of human blood plasma: a community-initiated position paper to develop accepted guidelines. *Journal of lipid research* 2018, *59* (10), 2001-2017. DOI: 10.1194/jlr.S087163 PubMed.

(8) Torta, F. Liquid Chromatography-Mass Spectrometry of Sphingolipids. In *Encyclopedia of Lipidomics*; Wenk, M. R., Ed.; Springer Netherlands, 2019; pp 1-6. DOI: 10.1007/978-94-007-7864-1_82-1.

(9) Committee for Medicinal Products for Human Use. ICH guideline M10 on bioanalytical method validation and study sample analysis. European Medicines Agency, Ed.; 2022.
